# Socioformative pedagogical practices and academic performance in students: Mediation of socioemotional skills

**DOI:** 10.1016/j.heliyon.2024.e34898

**Published:** 2024-07-20

**Authors:** Sergio Tobon, Edgar Fidel Lozano-Salmorán

**Affiliations:** a Centro Universitario CIFE, Cuernavaca, Mexico; b University of Colima, School of Veterinary Medicine and Animal Husbandry, Mexico

**Keywords:** Teaching, Socioformation, Pedagogical practices, Socio-emotional skills, Learning outcomes

## Abstract

This study analyzes the mastery level of educators regarding eight socioformative pedagogical practices and their impact on the academic performance of their students, aiming to identify mediating factors within a longitudinal design framework. A total of 282 university teachers from Ecuador participated, with instruments applied at two points in time: during the pandemic and at its end. Convenience sampling was employed. The results indicate an acceptable level in the development of socioformative pedagogical practices among participants, as well as in their socio-emotional skills. Regression analysis revealed the influence of various variables on socioformative pedagogical practices, such as socio-emotional skills, continuous training, academic degree, and economic level. The structural equation model established a positive association between socioformative pedagogical practices assessed during the pandemic and the academic performance of students in terms of achieving learning outcomes evaluated one year after the COVID-19 pandemic. It was also found that socioformative pedagogical practices are influenced by socio-emotional skills. The conclusion is that teachers possess a medium level in the development of their pedagogical practices but need to strengthen them to achieve an advanced level through continuous training and by obtaining higher academic degrees. Moreover, enhancing socio-emotional skills is essential for improved performance.

## 1 Introduction

The development of teaching competencies is key in universities. During the COVID-19 pandemic, these competencies were affected by the improvisation of online education in many universities, which also impacted motivation for learning and lowered the educational level [1]. These competencies are associated with better academic performance in learners, but the results are inconclusive, and research in this area is lacking [2]. It has also been established that students require new competencies to meet current and future challenges [3,4], such as teamwork, communication, leadership, and socio-emotional skills [5]. However, teacher education programs hardly address these areas [3,6], and diagnostic tools in teaching that address such aspects are scarce. The same could be said of socio-emotional skills, which are associated with the academic performance of university students, but little is addressed in teachers.

To develop teaching competencies, it is essential to evaluate them, particularly through self-evaluation by the teachers themselves [7,8]. Studies diagnosing this type of competencies have generally used scales [2,9], and it would be relevant to determine the impact of rubrics in measuring these competencies, which could increase reliability in the process and improve self-assessment among teachers. Moreover, these instruments have tended to focus on the tasks and activities expected in teaching, assessment, and the use of resources for learning, including planning (see, for example, [8]), and do not address the actions that should be implemented to transform education and precisely develop competencies in students, as is the challenge of several educational systems [10]. For example, the cited scales do not consider project work, solving environmental problems, developing complex thinking, etc.

In research over the last two decades, the concept of teaching competencies in higher education has prevailed [2]. This concept has gaps and a notable lack of clarity. For example, they tend to be assumed as a series of attributes of knowledge, skills, and values [11], but in practice, each aspect is worked on separately, which does not contribute to overcoming fragmentation in the training process. Therefore, the concept of socioformative pedagogical practices is proposed as an alternative, considering socioformation, a pedagogical model of Latin American origin, as an alternative to constructivism [12] and socioconstructivism. The new concept of socioformative pedagogical practices, unlike teaching competencies, focuses on acting on environmental problems with students, taking socio-emotional skills as a basis, without dividing between being, doing, knowing, and living together. If we focus on the new skills required in teaching, we will possibly help to strengthen them [13,14], and this is what is sought with socioformative pedagogical practices.

Based on the stated problem, the purposes of the present study are.

1. To determine the level of development of socioformative pedagogical practices in higher education teachers.
2. To establish the level of development of socio-emotional skills in teachers.
3. To determine if there are differences in pedagogical practices according to the following grouping variables: gender, age, years of experience, and academic degree.
4. To determine which factors are associated with greater development of pedagogical practices in university teachers through regression analysis.
5. To analyze whether socioformative pedagogical practices influence the achievement of learning outcomes after one year of follow-up through a longitudinal study and the use of structural equations.

## 2 Theoretical framework

### 2.1 Gaps in constructivism, socioconstructivism, and connectivism

In the past two decades, constructivism has dominated educational models in universities, as is the case in Ecuador. This pedagogical model focuses on student learning and the cognitive and affective processes involved through meaningful activities, differentiating itself from traditional education, which centers on teaching. The principles of constructivist teaching are [15]: 1) setting challenging objectives for each lesson with their evaluation criteria; 2) addressing goals with their criteria in students in an articulated manner; 3) addressing prior and future learning based on each lesson's objectives; 4) encouraging students to self-evaluate using evaluation criteria; and 5) applying formative evaluation and open-ended questions from the beginning in learning activities. This has been complemented by the vision of socioconstructivism, which integrates the social context to address learning, incorporating relationships with peers and others for skill development based on potential. Based on this, both constructivism and socioconstructivism have the following gaps: 1) they have individual learning as their paradigm, without considering sustainable social development; 2) they focus on the student and do not address group, school, and community learning processes, thus lacking a proposal for institutional transformation; and 3) they consider collaboration as a strategy for individual learning, not as a valuable process in itself.

To overcome the gaps in constructivism, connectivism has been developed, a new learning theory that emphasizes the importance of the digital environment, proposing that learning arises from connections within a network. This perspective holds that knowledge is created through interactions between nodes of information, including people, equipment, and technology. In this sense, not only does the individual learn—a central thesis of constructivism and socioconstructivism—but groups, organizations, machines, and networks also learn through the establishment of connections [[16], [17], [18], [19], [20], [21]]. The digital revolution, particularly the Internet, has radically changed our access to knowledge and the way we establish connections. From this perspective, connectivism emphasizes the need to explore information networks and maintain active links to facilitate continuous learning. Despite its influence, connectivism is not without criticism. It is reproached for focusing excessively on individual and machine learning without giving due importance to the formation of sustainable and inclusive communities. Additionally, it is criticized for its lack of proposals for a curricular integration that encompasses various disciplines and for the scarcity of specific didactic strategies that promote connectivist learning.

### 2.2 Socioformation: A new pedagogical model from Latin America

Socioformation is a new pedagogical model developed in Latin America, proposed by Tobón [22] and the collaborative work with teachers, educational authorities, experts, and researchers in a bottom-up and top-down construction process, taking into account the systematization of successful experiences in educational institutions. This new proposal aims to form citizens integrally to contribute to sustainable social development by addressing environmental problem situations (local, regional, national, and global) through collaboration, complex thinking, ethical life projects, knowledge management and co-creation, and digital technology (including artificial intelligence) [23]. To achieve this, students work on transversal projects with interdisciplinarity, applying continuous formative evaluation, and considering the millennium goals [24], in collaboration with social, community, business, and research organizations.

This new alternative differs from constructivism, socioconstructivism, and connectivism by focusing on integral formation and prioritizing transversal skills for living in society, while the other pedagogical models remain centered on learning, emphasizing cognitive processes. Secondly, socioformation assumes formation as a means to contribute to achieving sustainable social development. The other models assume learning as an end in itself, without focusing on sustainability. Thirdly, it seeks to transform environmental problems to improve living conditions through continuous problematization of reality and cooperation with social and business organizations through the implementation of interdisciplinary and transdisciplinary projects. Finally, it focuses on ethics and the development of universal values in every project, as well as socio-emotional skills. The other pedagogical models, in contrast, address this only when it is part of the learning objectives.

### 2.3 Problems with the concept of “teaching competencies”

In higher education in Ecuador, competency-based training has been gradually introduced based on the path established at the World Conference on Higher Education in 1998 [25]. This conference established competency-based teaching as a priority objective. Thus, some universities have been incorporating this approach through the transformation of their educational models [7,8]. However, competency-based training has various problems, such as: 1) it is oriented towards individual learning [26] and does not consider the actions of groups or communities; 2) it proposes an educational change by focusing on complex performance processes [8], but in practice, it has increased formalism, paperwork, and fragmentation of training due to the high number of subdivisions such as graduation profile competencies, subject competencies, topic competencies, learning outcomes, criteria, indicators, etc., [27]; and 3) it seeks the combination of attributes [28], but in practice, many teachers still address their components separately.

Teaching competencies are defined as complex qualities that teachers execute with students to achieve integral learning in the knowledge society and generate new actions based on priority skills. These skills are based on a well-founded education [6,29]. While this approach points to integral learning, it does not specify the nature of teaching competencies. This may become clearer when reviewing the teaching competencies proposed by several authors. A first approach classifies them into planning, communication, evaluation, methodology, digital skills, tutoring, leadership, and research [8]. Except for communication and leadership, the other competencies are typical of teaching functions and do not add a differential value to the daily tasks that every university teacher knows. This aligns with Vossen [30], who propose the following teaching competencies: learning planning, methodology, tutoring, evaluation, content mastery and innovation, and lifelong learning. In general, it can be observed that what is termed teaching competencies is actually a reference to functions or tasks that teachers must perform and do not represent a differential value, with some exceptions.

### 2.4 From teaching competencies to socioformative pedagogical practices

Due to the problems described with teaching competencies, socioformation proposes reconceptualizing the concept as pedagogical practices, a term that appears in the academic literature [[31], [32], [33], [34], [35]]. A practice represents a clear and straightforward action in reality, while competencies have remained in the notion of combining attributes and multiple subdivisions that affect their implementation. Thus, in socioformation, pedagogical practices are defined as integral actions that guide teachers, educational leaders, and others to help students contribute to sustainable development by solving problems in various contexts to improve living conditions [36] and contribute to sustainability [37,38].

Socioformative pedagogical practices have the following characteristics: 1) they focus on the integral action of teachers through collaboration, articulating universal values (responsibility, honesty, and respect), socio-emotional skills, critical thinking, professional identity, commitment, and creativity. In contrast, teaching competencies are assumed as tasks specific to teaching and generally focus on aspects inherent to teaching, such as planning, curricular design, tutoring, learning evaluation, and technology (see, for example, [30]); 2) they are not individual processes, like teaching competencies, but collaborative networked work processes mediated by teachers, involving other actors such as educational authorities, families, and community members; and 3) they do not focus on learning but on students learning to transform their environment by addressing problems with critical and complex thinking.

In socioformation, various pedagogical practices have been proposed, such as the 10 practices addressed in the SOCME-10 rubric study, which are: motivation and achievement of expected learning in students, concept learning through organizers and cases, problem-solving, formation of universal values and ethical life project, assertive communication, collaborative work, creativity development, transversal application, resource management, and formative and metacognitive evaluation [39]. However, there is a need for greater emphasis on improving living conditions and sustainable development. Therefore, the present research proposes the following practices: inclusion, problem-solving, socioformative evaluation, ethical life project, collaborative work, complex thinking, improvement of living conditions, and knowledge management.

## 3 Method

### 3.1 Type of study

Given the characteristics of the research, a repeated measures or longitudinal study was conducted [[40], [41], [42]], using four instruments to develop the research objectives at two different points in time, at the beginning and end of a university academic year [43]. Thus, the participating subjects were measured at two different moments [44,45] to analyze the achievement of socioformative pedagogical practices. The study met the criteria of longitudinal research in the context of repeated measures with the same sample of subjects over time [46], whose central criterion was to determine significant changes and differences among the subjects in the sample based on the variables involved in the study [47].

### 3.2 Participants

A total of 282 university teachers from various universities in Ecuador participated. Of these, 47.9 % were women, with an average age of 48.4 years (SD: 1.7 years) and an average of 7.65 years of teaching experience (SD: 5.9 years). Regarding economic conditions, 73.1 % reported having acceptable economic conditions, while 26.1 % stated they were low or very low. A total of 9.5 % lived in rural areas, 12.3 % in semi-urban areas, and 78.2 % in urban areas. In terms of education, 9.1 % had a bachelor's degree, 82.5 % had a specialization or master's degree, and 8.4 % had a doctoral degree.

### 3.3 Instruments

**Rubric for Socioformative Pedagogical Practices:** This instrument was created by CIFE [48] and aims to evaluate eight teaching practices through eight indicators. The rubric was developed following the socioformative taxonomy proposed by Tobon [49], which focuses on the process of acting in response to social and environmental context problems. Therefore, it serves as an alternative to Bloom's and Marzano's taxonomies, which are more content-focused. Each indicator contains five descriptors ranging from very low (1) to very high (5). In this study, a single factor was identified, explaining 49.3 % of the variance. All items showed factor loadings above 0.59, and the model fit was adequate [50]: X^2^/df = 2.29; GFI = 0.998; RMSAE = 0.020; RMR = 0.029; CFI = 0.971; NFI = 0.998; TLI = 0.989. The average extracted variance was 0.455. Both reliability measured by Cronbach's Alpha (0.89) and composite reliability (0.85) showed adequate levels.

**Rubric for Socio-emotional Skills:** Developed by Tobon [51], this rubric measures nine socio-emotional skills. Each skill is evaluated with five levels of mastery from very low to very high. The confirmatory factor analysis demonstrated two factors in this study: person-oriented skills (self-efficacy, self-regulation, self-motivation, self-esteem) and context-oriented skills (collaboration, resilience, decision-making, perseverance, social commitment, and empathy), explaining 46.6 % of the variance. All items had factor loadings above 0.61, and the final model showed good fit with X^2^/df = 1.88, CFI = 0.998, TLI = 1.04, GFI = 0.995, and RMSEA = 0.04, along with a composite reliability of 0.934 and an AVE = 0.566.

**Social Relations Scale:** This scale, created by CIFE [52], includes two dimensions: collaborative work with colleagues and social support received at school. Each scale has a Likert-type structure with five levels: 1 = very low, 2 = low, 3 = medium, 4 = medium-high, and 5 = very high. In this study, good levels of composite reliability were found: 0.91.

**Questionnaire on Sociodemographic Factors:** Developed by CIFE [53], this instrument collects participant data such as gender, age, place of residence, economic level, highest degree obtained, years of experience, and years of experience in the current position, among other information. Additionally, it inquired about the academic performance of students, such as the percentage of students who have not achieved expected learning outcomes, percentage of students lacking expected outcomes in reading, and percentage of students lacking expected outcomes in math.

### 3.4 Procedure

A cross-sectional study was conducted with participants selected by convenience. Teachers were invited to participate through social media announcements. The inclusion criteria were: 1) being a classroom teacher, 2) having assigned students, and 3) working in a public school. The instruments were applied using a Google Forms questionnaire. The process was developed in the following stages.

Phase 1 Determining the levels of socioformative pedagogical practices that teachers possess.

Phase 2 Analyzing differences in socioformative pedagogical practices based on gender, age, academic degree, and continuous training.

Phase 3 Determining factors associated with higher development of socioformative pedagogical practices through regression analysis.

Phase 4 Analyzing factors associated with students' academic performance to determine the impact of pedagogical practices using structural equation modeling.

#### 3.4.1 Statistical analysis

The study conducted the following statistical analyses.

1. **Normal Distribution Evaluation:** The normal distribution of the sample was assessed using skewness and kurtosis analysis, assuming a normal distribution when values were within the range of ±2.0 [[54], [55], [56], [57]].
2. **Descriptive Statistics:** Percentages of socioformative pedagogical practices levels were calculated and organized into low, medium, and high levels to establish the magnitude of skills. The mean was then tested for significant difference from the theoretical mean using a one-sample *t*-test or a similar non-parametric test [[58], [59], [60]]. Outliers were identified through distribution ranges (μ ± 1σ) to determine extremely low and high values based on the average means obtained from various variables, establishing a zone of normal behavior according to the standard deviation (σ) [61].
3. **Group Comparisons:** Differences in central variables concerning different grouping variables such as gender, age, place of residence, academic degree, etc., were tested using a *t*-test (two groups) or ANOVA (three or more groups) or with non-parametric tests. Significant differences in ANOVA were followed by post-hoc analysis using Tukey-Kramer due to differing group sizes. Effect sizes were added to comparisons when significant differences were found.
4. **Regression Analysis:** Variables associated with higher development of socioformative pedagogical practices were identified using multiple regression analysis with the stepwise procedure [58,62]. Total, measures of instruments and item measures of all remaining six variables (considering all sociodemographic variables) were included, except for students' academic performance variables, considering adjusted beta values and significance levels below 0.05. Effect size in this analysis was evaluated: Cohen's d values below 0.20 indicate no effect; values between 0.21 and 0.49 indicate a small effect; values between 0.50 and 0.79 indicate a moderate effect; and values above 0.80 indicate a large effect [63].
5. **Structural Equation Modeling:** This analysis determined the impact of variables on students' academic performance (variables 5.1, 5.2, 5.3, and 5.4) and their interactions, as well as the role of pedagogical practices (total and specific measures) and socio-emotional skills, along with other variables: collaborative work, social support, group size, and demographic variables. A p-value <0.05 was considered statistically significant [64]. Statistical analyses were performed using the free statistics software JASP v.0.17.3.0.

### 3.5 Ethical considerations

This study was reviewed and approved by School of Veterinary Medicine and Animal Husbandry of The University of Colima ethics committee, with the approval number: 1G.2.2/70600/095/2022, dated September 10, 2022. The following ethical criteria were adhered to in this study.

1. Participants were informed about the research, its purposes, and the instruments to be completed.
2. Participants gave written consent to participate in the study and complete the instruments, with the understanding that they could withdraw at any time without any questions.
3. Participants were informed about the confidentiality of their data and who would store it according to the Mexican Personal Data Protection Law [65].
4. Participants were assured that their data (email, IP, etc.) would not be sold or transferred to other organizations.

## 4 Results

### 4.1 Phase 1. level of development of socioformative pedagogical practices

Table 1 presents the level of development of socioformative pedagogical practices among the participants. For clearer interpretation, only three levels were considered: the low level integrates the very low and low levels; the medium level remains the same; and the high level sums up the medium-high and very high levels. It is observed that the highest percentages of socio-emotional skills are at the medium level, which also corresponds to the overall measure at the medium level.

**Table 1:** Percentages of socioformative pedagogical practices (n = 282).

Socioformative pedagogical practices	Level 1. Under	Level 2. Medium	Level 3. High	Average (+DS)	Asymmetry	Kurtosis	*t*-test (Theoretical mean: 3.0)	Cohen's d (Effect size)
Total value of the socioformative pedagogical practices rubric	16.667 %	37.254 %	46.079 %	3.51 (±0.001)	−0.298	−0.231	*t*: 56.002; *p < 0*.001	*0.7*
Inclusion	18.491 %	36.792 %	44.717 %	4.035 (±0.904)	−0.594	−0.389	*t*: 74.962; *p < 0*.001	*0.19*
Troubleshooting	17.359 %	43.396 %	39.**245 %**	4.032 (±0.824)	−0.290	−0.920	*t*: 82.186; *p < 0*.001	*0.21*
Socioformative evaluation	23.208 %	29.057 %	47.735 %	3.993 (±0.885)	−0.421	−0.602	*t*: 75.783; *p < 0*.001	*0.19*
Ethical life project	17.170 %	41.509 %	41.**321 %**	4.028 (±0.998)	−0.815	−0.024	*t*: 67.796; *p < 0*.001	*0.18*
Collaborative work	16.227 %	37.925 %	45.850 %	***4.152*** (±0.845)	−0.545	−0.754	*t*: 82.564; *p < 0*.001	*0.21*
Complex thinking	15.472 %	42.642 %	41.**887 %**	3.894 (±0.982)	−0.603	−0.230	*t*: 66.604; *p < 0*.001	*0.17*
Improvement of living conditions	15.661 %	33.585 %	50.755 %	3.851 (±1.047)	−0.486	−0.667	*t*: 61.796; *p < 0*.001	*0.16*
Knowledge management	13.207 %	30.377 %	56.415 %	3.929 (±0.944)	−0.649	0.027	*t*: 69.870; *p < 0*.001	*0.18*

*Note*. *t*-test Sig., p < 0.05; distribution ranges: μ + 1σ = 3.54 and μ - 1σ = 2.98.

A second analysis is also represented in Table 1. It can be observed that all teachers had a mean in all socioformative pedagogical practices significantly above the expected theoretical mean, which is 3.0. This value corresponds to the medium level evaluated on the scale and is considered satisfactory. Additionally, only one extreme value was found, significantly high compared to the others, related to collaborative work (m = 4.152). The effect size ranged from moderate to high for all considered variables, and the general measure of thinking skills had a high effect size.

### 4.2 Phase 2. differences in socioformative pedagogical practices based on different grouping variables

Using the Student's t-test, significant differences were found only between men and women regarding problem-solving and socioformative evaluation, with men showing higher levels in these skills (Table 2). There were no differences in the overall measure of socioformative pedagogical practices. The effect size was generally moderate or medium in the variables where significant differences were found.

**Table 2:** Differences in socioformative pedagogical practices between men and women.

Socioformative pedagogical practices	Comparison between groups			F (gl)	p value	η2
	Women	Men	Differences in averages			
Inclusion	4.03 (±0.90)	4.34 (±0.91)	−0.00	0.56	0.97	0.61
Troubleshooting	4.10 (±0.83)	3.96 (±0.81)	0.13	0.00	0.16	0.59
Socioformative evaluation	4.05 (±0.84)	3.93 (±0.91)	0.11	2.25	0.28	0.01
Ethical life project	4.02 (±1.02)	4.03 (±0.97)	−0.01	1.34	0.92	0.01
Collaborative work	4.20 (±0.85)	4.10 (±0.83)	0.09	0.88	0.36	0.01
Complex thinking	3.91 (±0.93)	3.87 (±1.02)	0.04	0.02	0.68	0.01
Improvement of living conditions	3.88 (±1.02)	3.82 (±1.06)	0.05	0.20	0.64	0.02
Knowledge management	3.92 (±0.94)	3.93 (±0.94)	0.09	1.10	0.95	0.01
General measure of socioformative pedagogical practices	3.59 (±0.89)	3.63 (±0.88)	−0.00	0.76	0.34	0.00

Note: *p < 0.05.

Table 3 presents the differences concerning years of experience. Three groups were established: 0–5 years, 6–18 years, and more than 18 years of experience. Significant differences were found only between the 0–5 years group and the group with more than 18 years of experience using the Tukey post hoc test. Teachers with more experience exhibited higher levels of socioformative pedagogical practices, and the effect size was moderate.

**Table 3:** Differences in socioformative pedagogical practices according to years of experience.

Socioformative pedagogical practice	Comparison between groups			F (gl)	p value	η2
	0–5	6–18 years	Over 18 years old			
Inclusion	3.966 (±0.98)	4.09 (±0.88)	4.00 (±0.81)	0.56	0.56	0.004
Troubleshooting	4.03 (±0.79)	4.03 (±0.83)	4.01 (±0.85)	0.00	0.99	0.005
Socioformative evaluation	3.82 (±0.94)	4.07 (±0.85)	4.03 (±0.83)	2.25	0.10	0.016
Ethical life project	3.88 (±1.03)	4.10 (±1.00)	4.05 (±0.89)	1.34	0.26	0.010
Collaborative work	4.10 (±0.82)	4.21 (±0.85)	4.05 (±0.85)	0.88	0.41	0.006
Complex thinking	3.89 (±0.97)	3.90 (±0.99)	3.86 (±0.97)	0.02	0.97	0.001
Improvement of living conditions	3.80 (±1.06)	3.85 (±1.04)	3.92 (±1.02)	0.20	0.81	0.001
Knowledge management	3.80 (±1.02)	3.97 (±0.90)	4.00 (±0.90)	1.10	0.33	0.008
General measure of socioformative pedagogical practices	3.59 (±0.89)	3.63 (±0.88)	3.63 (±0.88)	0.79	0.34	0.1

Note: *p < 0.05 Variables between which there were differences in the test are indicated.

Table 4 presents the differences concerning economic level. Three ranges were established: very low (sum of very low and low levels), medium, and high (sum of medium-high and very high levels). Differences were found in various socioformative pedagogical practices and the overall measure. The Tukey post hoc test revealed that teachers from high economic levels scored higher in problem-solving, ethical life project, and collaborative work, as well as in the overall measure, compared to other groups. However, only the differences in the overall measure of socioformative pedagogical practices showed a moderate effect size, which is relevant. The other comparisons had a low (non-relevant) effect size.

**Table 4:** Differences in socio-educational pedagogical practices according to the economic level of the family.

Socioformative pedagogical practices	Comparison between groups			F (gl)	p value	η2
	Low economic level	Average economic level	High economic level			
Inclusion	4.00 (±0.81)	4.03 (±0.91)	4.03 (±0.90)	0.01	0.98	0.075
Troubleshooting	4.00 (±0.81)	3.99 (±0.84)	4.09 (±0.79)	0.45	0.63	0.003
Socioformative evaluation	4.07 (±0.86)	3.94 (±0.90)	4.04 (±0.85)	0.44	0.64	0.003
Ethical life project	4.23 (±0.72)	3.96 (±1.05)	4.09 (±0.94)	0.76	0.46	0.005
Collaborative work	4.30 (±0.63)	4.07 (±0.86)	4.24 (±0.83)	1.51	0.22	0.011
Complex thinking	4.00 (±0.81)	3.84 (±1.01)	3.94 (±0.95)	0.40	0.66	0.003
Improvement of living conditions	4.23 (±0.83)	3.81 (±1.07)	3.85 (±1.02)	0.94	0.39	0.007
Knowledge management	3.69 (±0.94)	3.91 (±0.98)	3.97 (±0.88)	0.53	0.58	0.004
General measure of socioformative pedagogical practices	3.61 (±0.81)	3.62 (±0.85)	**3.71 (±** 0**.76)**	0.63	0.02^a^	**0.59**^b^

Note.

a p < 0.05.

b Moderate effect size.

Table 5 presents the differences in socioformative pedagogical practices by academic degree. Differences were found only at the doctoral level. The Tukey post hoc test revealed that teachers with a bachelor's degree have the highest development of socioformative pedagogical practices. However, the effect size was low (non-relevant).

**Table 5:** Differences in socioformative pedagogical practices according to academic grade level.

Socioformative pedagogical practice	Comparison between groups			F (gl)	p value	η2
	Bachelor's Degree	Master's Degree	PhD			
Inclusion	4.40 (±0.89)	4.02 (±0.89)	4.03 (±0.96)	0.41	0.66	0.003
Troubleshooting	4.40 (±0.89)	4.00 (±0.82)	4.11 (±0.80)	0.89	0.41	0.006
Socioformative evaluation	4.60 (±0.89)	3.97 (±0.88)	4.01 (±0.89)	1.25	0.28	0.009
Ethical life project	4.20 (±0.83)	4.06 (±0.97)	3.86 (±1.12)	0.89	0.40	0.006
Collaborative work	4.20 (±0.83)	4.14 (±0.86)	4.19 (±0.76)	0.08	0.92	0.005
Complex thinking	4.60 (±0.54)	3.84 (±0.96)	4.01 (±1.05)	1.96	0.14	0.014
Improvement of living conditions	4.40 (±0.89)	3.84(±1.04)	3.82 (±1.06)	0.70	0.49	0.005
Knowledge management	4.20 (±0.83)	3.90 (±0.94)	4.01 (±0.98)	0.53	0.58	0.004
General measure of socioformative pedagogical practices	3.60 (±0.83)	3.62 (±0.74)	3.65 (±0.75)	0.83	0.25	0.02

Note: *p < 0.05; **Low effect size.

Finally, Table 6 presents the differences with respect to pedagogical practices as a function of the number of hours of continuing education that teachers have had in the last five years. Differences were only found with respect to the general measure of pedagogical practices. In this regard, it was found that teachers with more than 120 h of training in the last two years presented higher levels of pedagogical practices.

**Table 6:** Differences in socioformative pedagogical practices according to hours of continuous education.

Socioformative pedagogical practice	Comparison between groups			F (gl)	p value	η2
	0–40 h	41–120 h	More than 120 h			
Inclusion	4.00 (±0.86)	4.04 (±0.96)	4.07 (±0.89)	0.17	0.84	0.001
Troubleshooting	3.98 (±0.81)	4.07 (±0.83)	4.05 (±0.83)	0.33	0.71	0.002
Socioformative evaluation	3.90 (±0.87)	4.03 (±0.91)	4.06 (±0.85)	0.86	0.42	0.006
Ethical life project	3.96 (±1.03)	4.08 (±1.00)	4.05 (±0.95)	0.38	0.67	0.003
Collaborative work	4.06 (±0.88)	4.18 (±0.77)	4.23 (±0.87)	0.98	0.37	0.007
Complex thinking	3.85 (±1.02)	3.92 (±1.01)	3.90 (±0.88)	0.89	0.89	0.0007
Improvement of living conditions	3.84 (±1.00)	3.79 (±1.12)	3.94 (±1.00)	0.49	0.87	0.609
Knowledge management	3.84 (±1.00)	3.99 (±0.94)	3.96 (±0.85)	0.62	0.53	0.004
General measure of socioformative pedagogical practices	3.61 (±0.81)	3.62 (±0.85)	**3.71 (±** 0**.76)**	0.59	0.02^a^	**0.59**^b^

Note.

a p < 0.05.

b Moderate effect size.

### 4.3 Phase 3. variables associated with teachers' socioformative pedagogical practices

The following section presents the multiple regression analysis to determine the variables associated with a higher degree of socioformative pedagogical practices among teachers in the first application. The association of various variables was analyzed, such as socio-emotional skills, collaborative work, social support, group size, and various sociodemographic variables, such as age, training time in the last five years, and training time in the last two years. This was done using the stepwise method. It was observed that different variables were associated with socioformative pedagogical practices, both as a general measure and specifically (Table 7). The effect size ranged from medium to large.

**Table 7:** Multiple linear regression with respect to variables associated with teachers' pedagogical practices.

Dependent variable	Predictor variable	Unstandardized coefficients	Standardized coefficients	t (Sig.)	95 % CI for B		Collinearity statistics	
		B (Desv Error)	Beta		Lower limit	Upper limit	Tolerance	VIF
Total measurement of socioformative pedagogical practices	Constant	−122.347 (6.815)		−17.952 (0.001)	−135.778	−108.916		
	General Measure of Social-Emotional Skills SE-9	0.144 (0.050)	0.178	2.400 (0.012)	0.09	0.032	0.902	1.009
	Training in the last 5 years	0.011 (0.082)	0.010	−0.134 (0.894)	−0.172	0.150	0.766	1.306
	Training in the last two years	0.027 (0.042)	0.046	−0.647 (0.518)	−0.001	0.056	0.773	1.293
	Academic degree	0.017 (0.170)	0.006	−0.101 (0.920)	−0.353	0.319	0.964	1.038
	Economic level	0.090 (0.099)	0.058	−0.906 (0.366)	−0.286	0.106	0.962	1.040
	Collaborative work	0.634 (0.080)	0.504	7.878 (<0.001)	0.475	0.793	0.966	1.035
	Group size	0.014 (0.016)	0.056	−0.867 (0.387)	−0.046	0.018	0.964	1.037

Source: self made.

### 4.4 Phase 4. structural equation modeling of student academic performance

**Fig. 1:**
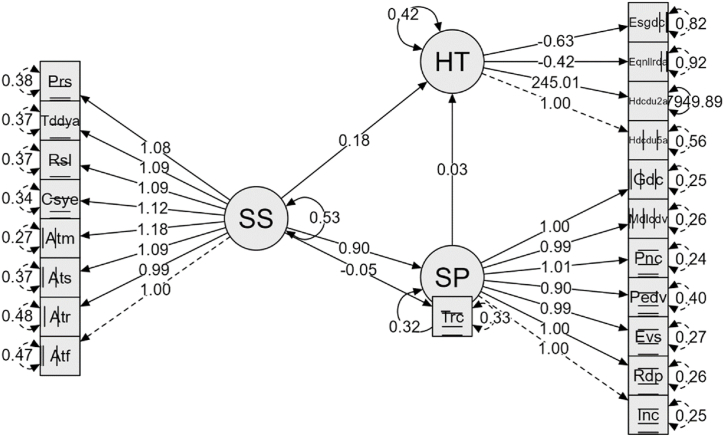
Standardized solution of the proposed model. SP = socioformative practices, SS = socio-emotional skills, HT = hours of didactic training: training in the last five years, training in the last two years, level of achievement of expected learning, level of achievement of learning in reading. *Inc*. inclusion, *Rdp*. Problem resolution, *Evs*. socioformative evaluation, *Pedv*. Ethical life project, *Trc*. Collaborative work, *Pnc*. Complex thinking, *Mdlcdv.* Improvement of living conditions, *Gdc*. Knowledge management, *Atf*. Self-efficacy, *Atr*. Self-regulation, *Ats*. Self-esteem, Atm. Self-motivation, Csye. Social commitment and empathy, *Rsl*. Resilience, *Tddya*. Decision making and autonomy, *Prs*. Perseverance, *Hdcdu2a*. Training hours during the last two years, *Hdcdu5a*. Training hours during the last five years, *Eqnllrda*. Students who did not achieve learning outcomes, *Esgdcl*. Students without a degree of reading comprehension.

As the final step, a structural equation modeling (SEM) analysis was conducted [66,67], to determine which variables impact the academic performance of students. The association of all variables from the first application (during the COVID-19 pandemic) with students' academic performance after one year ("Percentage of students who did not achieve the learning outcomes in their group or groups") was analyzed, and mediating variables were identified. The SEM model revealed that socioformative pedagogical practices are associated with students' academic performance (achievement of learning outcomes) but are influenced by socio-emotional skills. Additionally, the influence on achieving learning outcomes is mediated by continuous training. The proposed model is illustrated in Fig. 1, and the goodness-of-fit indices are detailed in Table 8.

**Table 8:** Model fit.

Statistician	Acceptance value	Model value	Decision
χ2	–	177.379	Acceptance
gl	–	141	Acceptance
Ratio χ2/gl	<3.0	1.903	Acceptance
IFC	>0.95	0.994	Acceptance
TLI	>0.95	0.993	Acceptance
GFI	>0.95	0.991	Acceptance
NFI	>0.95	0.882	Acceptance
PNFI	Close to 1	0.750	Acceptance
RMSEA	<0.08	0.061	Acceptance

## 5 Discussion

This study shows that higher education teachers tend to self-evaluate their pedagogical practices above the medium level, without reaching a medium-high or high level (which would be excellence). This aligns with findings from other studies [38,68]. Teachers do not exhibit low levels but neither do they show high levels in their socioformative pedagogical practices, indicating an opportunity for improvement. A key finding is that teachers reported a medium level in their socioformative pedagogical practices, which represents a valuable opportunity for student training, suggesting a moderate guarantee that these practices can impact student learning.

The pedagogical practices with the highest development were inclusion, problem-solving, socioformative evaluation, strengthening the ethical life project, and students' collaborative work. This is consistent with some previous studies that have found similar results, such as Coaquira [38]. Regarding pedagogical practices with an acceptable level of development, this research identified critical thinking, improvement of living conditions, and knowledge management, findings that have also been reported in other studies. The difference with these studies is that this research applied four instruments, which constituted a key and important element of analysis, introducing a different perspective on evaluating teaching competencies in Latin America.

An advancement in the implementation of some elements of the socioformation pedagogical model can be observed, either explicitly or implicitly, through a greater development of inclusive pedagogical practices focused on problem-solving based on collaborative work, which are the essence of this Latin American perspective. This study did not find differences between men and women regarding socioformative pedagogical practices. Studies have reported diverse information on this matter, suggesting that in the Ecuadorian context, gender, age, and economic level do not constitute determining factors for the development of teaching competencies, emphasizing the importance of socio-emotional attributes in this profession.

Furthermore, this research found that teachers with more years of work experience exhibit higher levels of pedagogical practices. Similarly, those with a doctoral degree also tend to have higher levels of socioformative pedagogical practices, indicating that teaching trajectory combined with continuous training is a key factor for pedagogical development. Additionally, academic degree reinforces confidence for professionals to reflect on their practice and determine, adjust, and reconceptualize their didactic spectrum in the classroom.

What factors are associated with a higher level of socioformative pedagogical practices in teachers? Multiple regression analysis found that socio-emotional skills are strongly associated with higher levels of pedagogical practices. This may be because better emotion management is associated with higher levels of mental health and better work performance among teachers [69,70]. Socio-emotional skills also help achieve better school outcomes [71,72] and improve teaching processes [73].

It was also found that more hours of continuous training were associated with a greater development of socioformative pedagogical practices. Studies show that teacher training is associated with better teaching strategies, better evaluation practices, and more support [74]. Likewise, more years of professional experience correlated with greater development of socioformative pedagogical practices, consistent with other studies showing that more professional experience in teaching leads to better class and group management [75].

A higher degree of socioformative pedagogical practices was also associated with greater collaborative work in the school, as reported by other studies [76]. Increased collaborative work may help identify and improve errors in teaching practice, as well as coordinate efforts in the school to enhance didactic strategies. Furthermore, this research found that socioformative pedagogical practices were associated with better student academic performance, specifically regarding the achievement of expected learning outcomes and reading. Other studies have also reported that pedagogical practices are associated with better academic performance [77,78]. A very important aspect of this study was that the impact of socioformative pedagogical practices on academic performance is mediated by teachers' socio-emotional skills.

Some limitations of this study include: 1) the socioformative pedagogical practices are circumscribed to a specific population and context, so the study presents results that correspond to a localized group of people and, while they serve as a reference, they cannot be generalized to all higher education teachers; 2) the statistical analysis behaved desirably, yet only conventional reporting was provided; other authors or study types might delve deeper; and 3) the structural equation model was closest to the goodness-of-fit values; however, for research purposes, it left out the projection of residual covariances.

This study aimed to surpass the concept of teaching competencies prevalent in much research, inspired by authors like Perrenoud [79], and instead proposed the concept of pedagogical practices, finding evidence of their association with the achievement of learning outcomes. However, the findings must be interpreted in the context in which the research was conducted, namely some universities in Ecuador, with a non-probabilistic sample.

Competency evaluation should involve students, intensifying feedback and co-responsibility, promoting teacher development, planning, and converting the culture of evaluation into research and clarifying reference that promotes integral innovation [80,81]. It highlights the significance and complexity of evaluation by and for competencies among teachers from their usual work scenarios and the impact on their professional development [82].

## Data availability statement

The data associated with this study have not been deposited in a publicly accessible repository. However, the data will be made available from the corresponding author upon reasonable request.

## CRediT authorship contribution statement

**Sergio Tobon:** Validation, Supervision, Project administration, Methodology, Data curation. **Edgar Fidel Lozano-Salmorán:** Writing – review & editing, Writing – original draft, Software, Investigation, Formal analysis, Data curation, Conceptualization.

## Declaration of competing interest

The authors declare that they have no known competing financial interests or personal relationships that could have appeared to influence the work reported in this paper.
